# Temperature effects on vanadium speciation and adsorption to biochar alone and biochar–metal oxide nanoparticle composites

**DOI:** 10.1002/jeq2.70139

**Published:** 2026-01-22

**Authors:** Dileep Singh, Srimathie Indraratne, Bhavya Anil, Melissa Haak, Darshani Kumaragamage, Doug Goltz

**Affiliations:** ^1^ Department of Environmental Studies and Sciences The University of Winnipeg Winnipeg Manitoba Canada; ^2^ Department of Chemistry The University of Winnipeg Winnipeg Manitoba Canada

## Abstract

Vanadium (V) is a potentially toxic metal widely distributed in the environment. This study investigates temperature effects on V adsorption and speciation in biochar (BC) and BC–metal oxide composites under conditions relevant to contaminated soils in temperate climates. While BC and metal oxide nanoparticles can individually immobilize V, limited information exists on temperature effects. This study investigates V adsorption and surface characteristics of BC alone and BC combined with iron (Fe), aluminum (Al), and titanium (Ti) oxide nanoparticles (BC: oxides at 5:1 ratio) at warm (22°C) and cold (4°C) temperatures. V adsorption was conducted at pH 7.5 using concentrations from 0 to 40 mg L^−1^. Visual MINTEQ modeling software was used to predict dissolved V species at experimental conditions. Surface characteristics were examined using scanning electron microscopy‐energy dispersive X‐ray spectroscopy (SEM‐EDS) and Fourier transform infrared spectroscopy. Adsorption data were fitted to the Freundlich (*r* ^2^ ∼0.99) and Langmuir (*r* ^2^ = 0.66–0.96) isotherms. Maximum adsorption capacity followed the order: BCAl‐cold = BCAl‐warm > BCTi‐cold > BCTi‐warm = BC‐cold = BC‐warm > BCFe‐warm = BCFe‐cold. Predicted orthovanadate (%) was higher at warm temperatures. H_2_VO_4_^−^ was the dominant species at pH 7.5 under both temperatures. Microaggregates were observed in BCAl and BCTi, indicating greater surface area than BC or BCFe. SEM‐EDS showed V and Al enrichment on BCAl surfaces suggesting the inner‐sphere complexes between Al–oxygen (O) and H_2_VO_4_^−^. These results offer mechanistic insight into V adsorption on BC–nano‐oxide composites under varying climatic conditions and support their potential use in remediating V‐contaminated soils.

## Plain Language Summary

Vanadium is a toxic metal that can pollute soil and water, especially near factories and mines. It is important to find safe and effective ways to stop it from spreading. One option is biochar, a black, charcoal‐like material made from plants, which can trap harmful metals. Another option is using tiny metal particles, called nanoparticles, but using too many can be expensive and risky for the environment. This study tested a new idea: mixing small amounts of metal nanoparticles with biochar to remove vanadium more safely and effectively. The researchers tested this mix at cold and warm temperatures, like those found in northern climates. They found that the mix with aluminum particles (called BCAl) was the most effective. It held onto vanadium better than the others. Using special imaging tools, the researchers saw that vanadium was sticking to the aluminum areas on the biochar. The study also showed that temperature affects how vanadium moves and reacts in cold and warm temperatures.

**Abbreviations**
BC: biochar
EDS: energy dispersive X‐ray spectroscopy
FTIR: Fourier‐transform infrared spectroscopy
SEM‐EDS: scanning electron microscopy‐energy dispersive X‐ray spectroscopy

## 1 INTRODUCTION

Vanadium (V) is a transition metal element and one of the most abundant trace elements in the Earth's crust (Wnuk, 2023). Anthropogenic activities such as mining, smelting of V‐minerals, stone coal processing, and fossil fuel combustion have led to elevated V levels in soils (Haak & Indraratne, 2023; Yang et al., 2017). With its increasing use in energy storage, steel alloys, and industrial catalysts, V has gained attention as an emerging environmental contaminant (Gao et al., 2022). In the environment, V exists in multiple oxidation states, predominantly as V(III), V(IV), and V(V) (Larsson et al., 2013). Speciation plays a critical role in both the mobility and toxicity of V, with reduced forms [e.g., V(III)] typically forming stable surface complexes or precipitates, while oxidized forms persist in the aqueous phase (L. Chen et al., 2021; Shaheen et al., 2019). Among them, V(V) is the most mobile and toxic, existing as oxyanions such as H_2_VO_4_^−^ and HVO_4_^2^^−^, which weakly bind to soil minerals and are prone to leaching into groundwater (L. Chen et al., 2021; Yang et al., 2017). Global assessments reveal elevated V concentrations in industrial soils in regions such as China, South Africa, and the western United States, often exceeding ecological and human health thresholds (Ghosh et al., 2015; Yang et al., 2017). Acute and chronic exposure to V(V) has been associated with nephrotoxicity, oxidative stress, and DNA damage (Parveen et al., 2025). Concentrations in V‐contaminated soils may exceed 600 mg kg^−1^, significantly above background levels (<1–200 mg kg^−1^) (Aihemaiti et al., 2018; Teng et al., 2006).

Adsorption is widely regarded as a cost‐effective and scalable method for mitigating metal contamination in soils (Jiang et al., 2019). Biochar (BC), a carbon (C)‐rich material produced via pyrolysis, is effective in immobilizing trace metals and improving soil health (Kończyk et al., 2022). Additionally, metal (hydr)oxides, such as iron (Fe), aluminum (Al), and titanium (Ti), exhibit a high affinity for oxyanion contaminants like V(V) (Haak et al., 2024; Shaheen et al., 2019). However, the use of metal oxide nanoparticles alone raises concerns due to their high cost and uncertain fate in soils, including plant uptake (Ukwattage & Zhiyong, 2025). This study employs a composite approach, mixing BC with nanoparticles at a 5:1 ratio to enhance adsorption while minimizing environmental risk.

Temperature influences both V speciation and adsorption behavior, yet prior studies were predominantly conducted under warm temperature ranges (e.g., 25–60°C), overlooking seasonal conditions typical of contaminated soils in temperate regions (Jung et al., 2018; Zhao et al., 2022). Sorption of V is known to decrease in alkaline soils, common in temperate zones, due to reduced surface affinity and competition from anions like phosphates (Abernathy et al., 2022; Haak et al., 2024). However, the interactive effects of temperature and speciation on V adsorption to BC–nano‐oxide composites remain poorly understood.

This study addresses a key knowledge gap by evaluating V adsorption and speciation in BC and BC–metal oxide composites (Fe, Al, and Ti) under environmentally relevant soil temperatures (4°C and 22°C), representative of spring and summer conditions in northern temperate regions. While most studies focus on aqueous systems or elevated temperatures, limited research has explored V behavior in BC‐based systems under lower temperature regimes typical of field‐contaminated soils. We hypothesize that combining BC with selected metal oxide nanoparticles at a low 5:1 BC:nano‐oxide ratio enhances V adsorption capacity and modifies surface interactions in a temperature‐dependent manner, while minimizing potential environmental risks. The chosen nano‐oxides of Fe, Al, and Ti reflect common soil mineral phases with a known affinity for vanadate species. Through adsorption isotherms, speciation modeling, and surface characterization, we investigate the mechanisms governing V retention to inform remediation strategies for contaminated soils under environmentally relevant conditions.

## 2 MATERIALS AND METHODS

Sodium orthovanadate (Na_3_VO_4_, 99.9%) and nano‐oxides of iron (Fe_3_O_4_), aluminum (Al_2_O_3_), and titanium (TiO_2_) were purchased commercially (Sigma‐Aldrich). BC made from 100% wood was purchased from Canadian Agrichar (Char+) and had a 70% carbon and 30% ash ratio. Detailed Fourier‐transform infrared spectroscopy (FTIR) characterization of the wood BC used in this study has been previously published in Haak et al. (2024). Briefly, the spectra confirmed the presence of phenolic, alcoholic, aromatic, and polysaccharide‐derived functional groups. The surface morphology of BC is illustrated in Figure S1, and the total elemental composition is given in Table S1. Product specification data for Fe_2_O_3_, Al_2_O_3_, and TiO_2_ nanoparticles are provided in Table S2. All experiments used ultrapure water (Milli‐Q; 18 MΩ cm). A 1000 mg V L^−1^ stock solution was prepared with Na_3_VO_4_.

### 2.1 Determination of adsorption capacities

Vanadium adsorption isotherm experiments were conducted for BC and nano‐oxides‐combined biochar mixtures (BCFe, BCAl, and BCTi), prepared by mixing BC and metal oxide nanoparticles at 5:1 (w/w) ratio. The BC and nano‐oxide mixtures were prepared by lightly grinding both amendments using a mortar and pestle, followed by intermittent shaking in a 50‐mL Falcon tube for approximately 30 min to ensure homogeneous mixing. These isotherms were conducted at two temperatures mimicking summer (warm; 22°C) and spring (cold; 4°C) conditions. Working solutions of V at concentrations of 0.5, 1, 5, 10, 20, 30, and 40 mg L^−1^ were prepared to simulate low to moderately contaminated soil environments. This range aligns with those used in previous soil adsorption studies aimed at evaluating the performance of BC and its derivatives under environmentally relevant conditions (Dong et al., 2024; Fan et al., 2020; Kończyk et al., 2022). Each solution was buffered at pH 7.5 with 2 mL of 1 M piperazine‐N,N′‐bis 2‐ethanesulfonic acid (PIPES) buffer to reach a final concentration of 0.01 M PIPES. Adsorption experiments were conducted at a controlled pH of 7.5, selected to represent the alkaline conditions of the target V‐contaminated soil system and ensure relevance to subsequent soil application. The ionic strength of the solution was adjusted to 0.01 M NaNO_3_. For each treatment, 0.15 g of the adsorbent and 30 mL of the prepared working solutions were added to labeled Falcon tubes. The solutions were shaken for 24 h and then centrifuged at 5000 × *g* for 20 min. A 24‐h equilibration time was used based on our prior adsorption edge study (Haak et al., 2024); however, temperature‐dependent kinetics were not assessed and are noted as a limitation. The supernatant was filtered through a 0.45 µm filter and stored in scintillation vials at 4°C until analysis. The equilibrium V concentrations in the supernatant were determined by inductively coupled plasma‐optical emission spectrometry (Thermo iCAP 6500 Duo). The same adsorption experiment was repeated at 4°C in a walk‐in cooler. The prepared solutions and adsorbents were stored in a cooler for 24 h before the start of the adsorption study. Freundlich and Langmuir isotherms were fitted to the data, and isotherm parameters were calculated (Foo & Hameed, 2010). Conformity to the Freundlich‐type adsorption isotherm was tested using the equation *Q*_eq_ = *K_F_* × *C*_eq_^(1/^ *^n^* ^)^; where *Q*_eq_ is the amount of V adsorbed by the amendment (mg kg^−1^), *C*_eq_ is the concentration of V in the equilibrium solution (mg L^−1^), and *K_F_* and 1/*n* are the empirical constants. Conformity to the Langmuir‐type isotherm was tested using the linear equation: *C*_eq_/*Q*_eq_ = 1/(*K_L_* × *Q_M_*) + *C*_eq_/*Q_M_*, where *Q_M_* is the maximum amount of V that can be sorbed in a monolayer (mg kg^−1^) and *K_L_* is the equilibrium constant, which represents the intensity of the adsorption isotherm (L kg^−1^).

**Core Idea**

- Combining biochar (BC) with metal oxide nanoparticles enhanced surface adsorption sites.
- BC + aluminum (Al) oxide composites exhibited higher vanadium (V) adsorption capacity than BC alone.
- V adsorption fitted both Langmuir and Freundlich isotherm models.
- The proportion of predicted V species varied with warm and cold temperatures.
- Adsorption capacity varied under cold (4°C) and warm (22°C) conditions.

### 2.2 Thermodynamic modeling

The geochemical equilibrium speciation modeling software, Visual MINTEQ 3.1 (Gustafsson, 2013) was used to estimate the percentage of each ortho‐ and polyvanadate species at pH 7.5 in each V concentration level for warm and cold temperature conditions separately. The concentrations of V(V) used in the adsorption experiment were 0.5, 1, 5, 10, 20, 30, and 40 mg L^−1^ (9, 19, 98, 196, 392, 588, and 785 µM of V). The input parameters used for the model were 0.01 M Na^+^, 0.01 M NO_3_^−^, and pH 7.5.

### 2.3 Characteristics of vanadium–amendment complexes

Solid samples of each adsorption study (BC, BCFe, BCAl, and BCTi) at the highest concentration (40 mg L^−1^) were dried in air for several days, followed by oven drying at 85°C for 24 h. The post‐sorption composites were washed with 3–5 mL of deionized water (Milli‐Q) before drying to remove residual salts and buffer components. The dried amendment‐V solid samples were then ground to <150 µm in an agate mortar and pestle. FTIR was performed on solid samples using an Alpha II Platinum FTIR spectrometer (Bruker D8 Advance). Transmittance spectra were acquired by mixing the samples in KBr pellets. For each sample, 16 spectra were acquired with 4 cm^−1^ resolution (400–4000 cm^−1^).

Scanning electron microscopic (SEM) images were used to visualize BC‐V and BC‐nano‐oxide‐V surfaces. A small amount of powdered sample was mounted and spread evenly using a toothpick on carbon tape pasted on the sample holder. Everhart–Thornley Detector in the FEI Quanta 650 environmental SEM was used for imaging. SEM images were obtained at several locations per stub, and the percent elemental abundance for selected elements was determined. The Octane Super SDD detector by EDAX and the Software TEAM were used for energy dispersive X‐ray spectroscopy (EDS) elemental analysis. Secondary electron or backscattered electron images were collected at 15 keV using a range of magnifications (100–2500) to observe surface properties as well as spatial mapping of the elemental composition of BC and BC–nano‐oxide mixtures.

### 2.4 Statistical analysis

Linear and nonlinear regression analyses were performed to determine the significance of the relationship of Langmuir and the Freundlich isotherms, separately. The assumptions of normality and homogeneity of variance for analysis of variance (ANOVA) were evaluated using residual analysis (via *Q–Q* plots) and Levene's test, respectively. Before performing ANOVA, the data for *Q_M_* and *K_F_* were log‐transformed (as assumptions were not met), while the data for 1/*n* and *K_L_* were analyzed without transformation. As the interaction effect was found to be significant, Tukey's post hoc tests were conducted for all combinations of temperature and adsorbent. Root mean square error (RMSE) and Akaike information criterion (AIC) were used to evaluate models, while the adjusted coefficient of determination (adjusted *r* ^2^) was used to evaluate the conformity to isotherms. The significance level was *p* < 0.05.

## 3 RESULTS

### 3.1 Vanadium adsorption capacities at warm and cold temperatures

**FIGURE 1:**
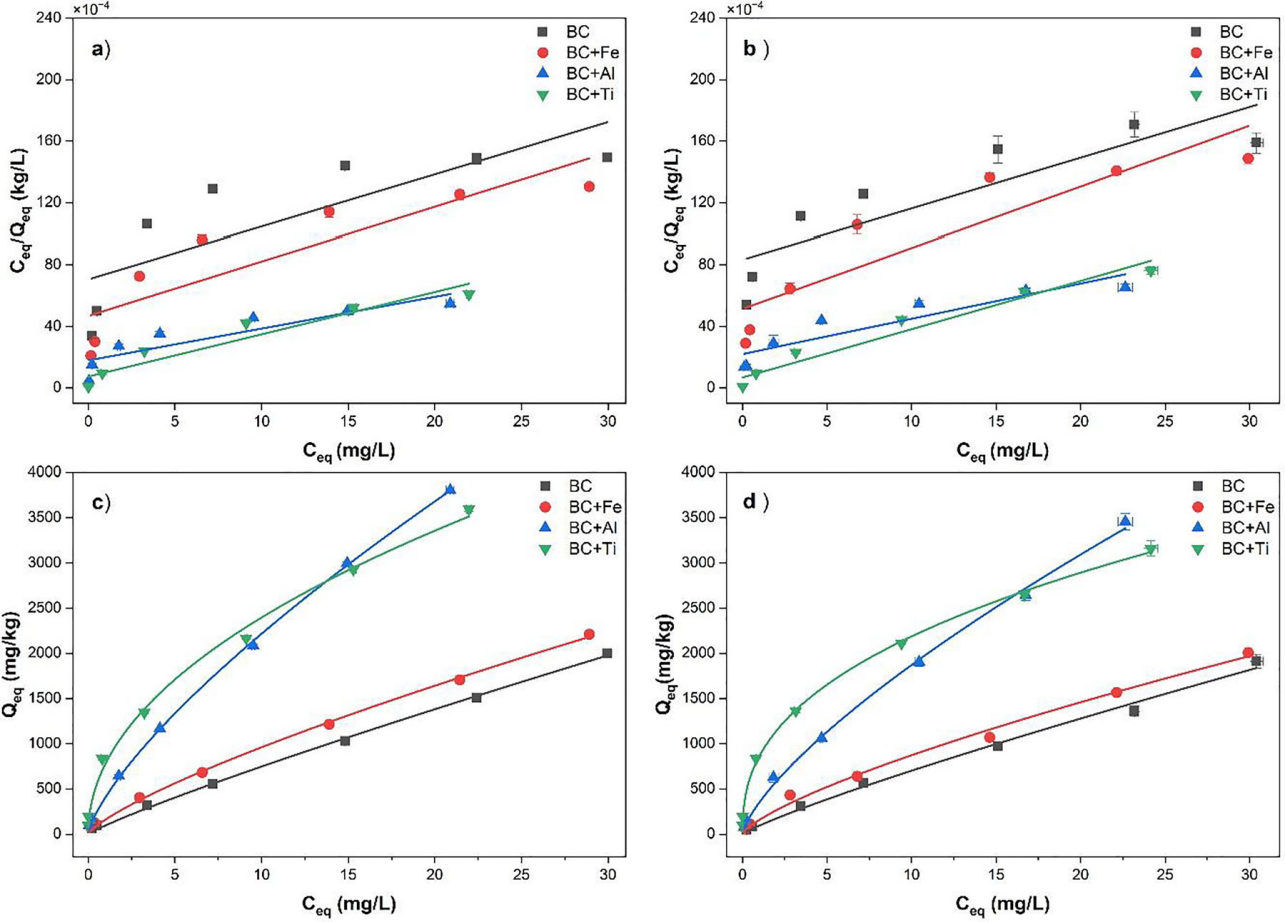
Linear form of Langmuir (a and b) and Freundlich (c and d) isotherms at cold (4°C; a and c) and warm (22°C; b and d) temperatures (BC = biochar; Fe, Al, and Ti were the metal oxide nanoparticles).

For the two isotherms tested, the Freundlich isotherm showed lower AIC criterion and RMSE than that of Langmuir (Table 1) for each adsorbent, confirming the best fit for the Freundlich isotherm (Dávila‐Jiménez et al., 2014). The conformity to both Langmuir and Freundlich isotherms was significant, the Freundlich isotherm being superior (*r* ^2 ^= 0.99; *p* = 0.001) to the Langmuir (*r* ^2^ from 0.66 to 0.95; from *p* = 0.05 to *p* = 0.001) (Figure 1). The treatment, temperature, and interaction effects were significant for both Langmuir (*Q_M_* and *K_L_*) and Freundlich (*K_F_* and 1/*n*) parameters (Table S3). The highest adsorption capacity (*Q_M_*) was observed in BCAl, and the temperature effect was non‐significant. In BCTi, the cold temperature *Q_M_* was significantly higher than the warm temperature *Q_M_*. BC and BCFe did not show a significant temperature effect. The Langmuir constant *K_L_*_,_ followed the same trend as *Q_M_*. The maximum adsorption (*Q_M_*) of vanadate can be arranged in the order of BCAl‐cold = BCAl‐warm > BCTi‐cold > BCTi‐warm = BC‐cold = BC‐warm > BCFe‐warm = BCFe‐cold (Table 1).

**TABLE 1:** Adsorption isotherm parameters for adsorbents at two temperatures.

	Langmuir equation *C*_eq_/*Q*_eq_ = 1/(*K_L_* × *Q_M_*) + *C*_eq_/*Q*_M_				
Adsorbent	*Q_M_* (mg kg^−1^)	*K_L_* (L kg^−1^)	*r* ^2^	RMSE	AIC
BC (22°C)	3029C	0.040F	0.759^*^	127	74
BCFe (22°C)	2528D	0.077DE	0.823^**^	163	78
BCAl (22°C)	4799A	0.081CD	0.840^**^	207	81
BCTi (22°C)	3179C	0.460A	0.959^***^	294	86
BC (4°C)	2947C	0.048EF	0.664^*^	150	77
BCFe (4°C)	2840CD	0.075DE	0.781^**^	172	79
BCAl (4°C)	4835A	0.115C	0.805^**^	275	85
BCTi (4°C)	3630B	0.370B	0.922^***^	377	90

	Freundlich equation *Q*_eq_ = *K_F_* × *C*_eq_^(1/^ *^n^* ^)^				
Adsorbent	*K_F_*	1/*n*	*r* ^2^	RMSE	AIC
BC (22°C)	96F	0.865A	0.993^***^	62	65
BCFe (22°C)	159D	0.740C	0.994^***^	62	65
BCAl (22°C)	353C	0.725C	0.998^***^	64	65
BCTi (22°C)	866A	0.403E	0.999^***^	38	58
BC (4°C)	98F	0.885B	0.998^***^	36	57
BCFe (4°C)	163D	0.772D	0.998^***^	37	57
BCAl (4°C)	412B	0.731D	0.999^***^	36	57
BCTi (4°C)	783A	0.486E	0.996^***^	98	71

Abbreviations: AIC, Akaike information criterion; BC, boichar; RMSE, root mean square error.

*, ** and *** followed by *r* ^2^ indicate significant at *p* < 0.05, 0.01, and 0.001, respectively; different letters indicate a significant difference at *p* < 0.05).

The Freundlich isotherm model is considered to be appropriate for describing both multilayer sorption and sorption on heterogeneous surfaces (Coles & Yong, 2006). The Freundlich constant, *K_F_* showed the highest value in BCTi and the lowest value in BC (Table 1). The temperature effect was significant in BCAl (Table S3), where cold temperature *K_F_* was higher than warm temperature *K_F_*. The value of the Freundlich parameter, 1/*n*, was between 0.40 and 0.88, suggesting that the adsorption was favored on all surfaces at both warm and cold temperatures. Adsorption on heterogeneous surfaces was more realistically expressed by the Freundlich model, where the amount adsorbed increased as the equilibrium concentration of the pollutant increased (Söğüt & Gülcan, 2023). The Freundlich heterogeneity parameter 1/*n* also changed significantly among treatments, with BCTi having the lowest and BC having the highest, indicating the highest heterogeneity in BCTi. The Freundlich isotherm is linear if 1/*n* = 1, and as 1/*n* decreases, the isotherm becomes more nonlinear (Coles et al., 2006). The temperature effect on 1/*n* was non‐significant in BCTi, yet in all other treatments’ warm temperature had a significant value than cold temperature. Hence, we can conclude, according to the adsorption parameters, that the amendments BCAl and BCTi were superior adsorbents for V in both warm and cold temperatures. The temperature effects on adsorption parameters were not consistent.

### 3.2 Vanadium speciation with varying concentrations in warm and cold temperatures

**FIGURE 2:**
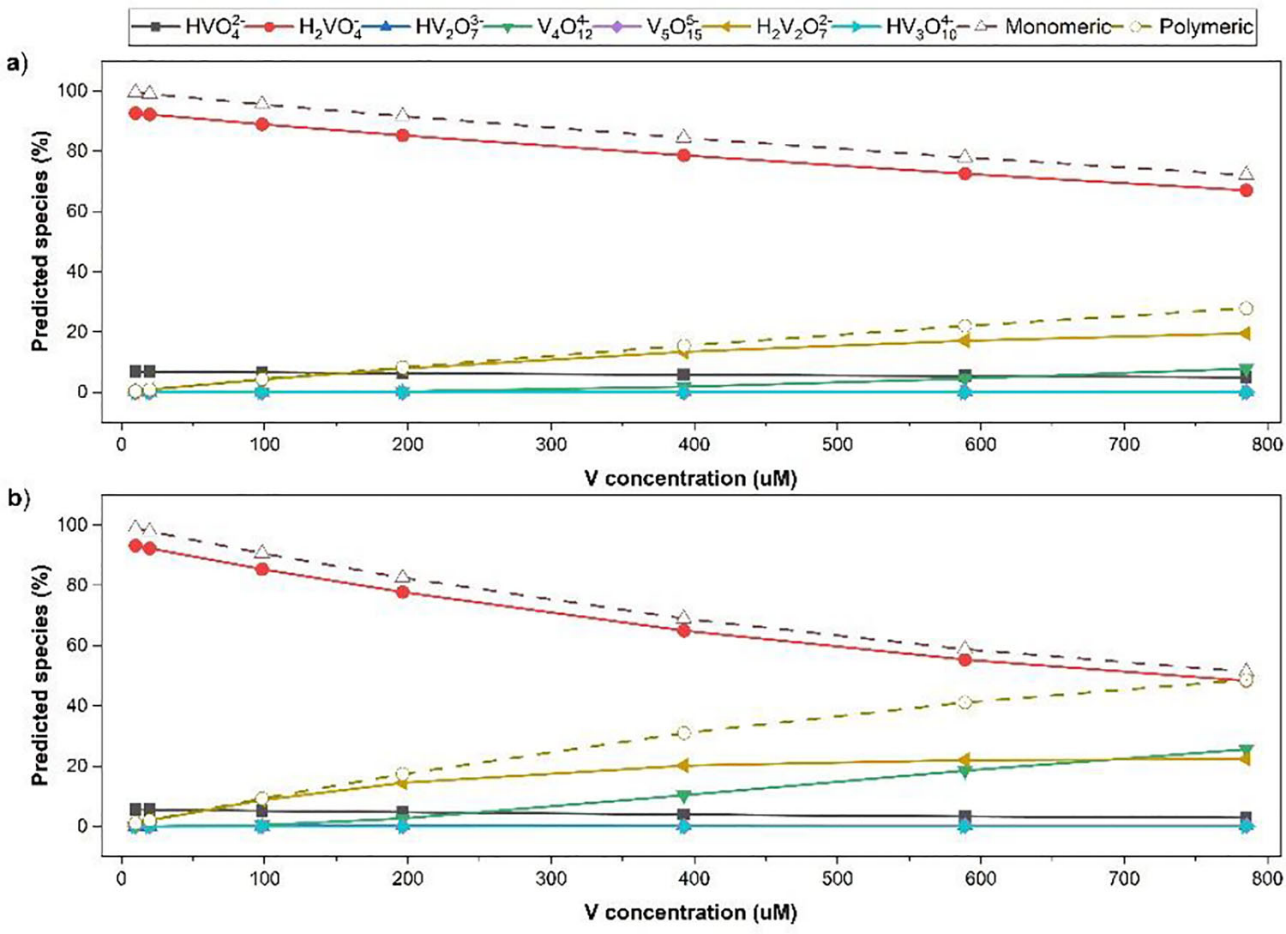
Percentage of predicted (MINTEQ) V species (total of adsorbed and dissolved) at pH 7.5 from total concentration of V added at 22°C (a) and 4°C (b) temperatures.

Vanadium can exist in different forms in aqueous systems, according to the speciation diagram for V (Figure 2). At pH 7.5, the V species changes with increasing concentration from 0.5 mg L^−1^ (9.8 µM) to 40 mg L^−1^ (785 µM) as given in Figure 2, at both temperatures. In warm temperature (22°C), the predicted percentage of orthovanadate species of HVO_4_^2−^ and H_2_VO_4_^−^ declined from 6.9% to 4.9% and 93% to 67%, respectively, with increasing V concentrations (Figure 2a). In addition to the decline in orthovanadate species, percentage of polyvanadates increased under warm temperatures (the percentage of polyvanadates of V_4_O_12_^4−^ and H_2_V_2_O_7_^2−^ increased from 0% to 7.9% and 0% to 19.6%, respectively), with increasing V concentrations. Orthovanadate decline was more drastic at 4°C in comparison to warm temperatures declining to final percentages of 3% and 48% in HVO_4_^2−^ and H_2_VO_4_^−^, respectively (Figure 2b). Similarly, polyvanadate percentages increased to 25.4% and 22.6%, respectively, for V_4_O_12_^4−^ and H_2_V_2_O_7_^2−^ at cold temperature. This difference in the predicted composition of the dissolved V species at cold and warm temperatures may likely explain the differences in adsorption capacities of the different amendments.

### 3.3 Spectroscopic differences of vanadium–amendment complexes

**FIGURE 3:**
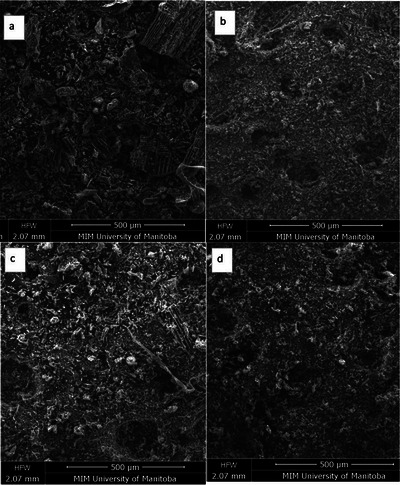
Secondary electron or backscattered electron (BSE) images collected at 15 keV at 100 magnification for amendments of biochar (BC, a), BCFe (b), BCAl (c), and BCTi (d).

SEM‐EDS was used to examine the surface morphology of the amendments, V distributions, and their elemental associations with the amendments. Representative images from the SEM studies are shown in Figure 3. The SEM experiments revealed that the size of the particles in BCAl and BCTi was smaller, suggesting smaller microaggregates than in BC and BCFe (Figure 3). The sizes and shapes of the pores also differed in BCAl and BCTi, resulting in the enhanced specific surface area and V adsorption capacity.

The weight percentage of C was highest in BC (70 ± 0.4), while BCAl (59 ± 1.3), BCFe (55 ± 0.1), and BCTi (51 ± 0.9) showed lower percentages (Table S2). Relative to BC, the oxygen (O) weight percentage increased slightly in BCFe and noticeably in BCAl and BCTi mixtures. Elemental composition showed elevated percentage of Fe, Al, and Ti in BCFe, BCAl, and BCTi, respectively. Al was present in all treatments (Al < 1%), while Fe was present in BC + nano‐oxide (Fe < 1%) and Ti only in BCTi.

Element concentrations (weight %) in amendment–V complexes after being subjected to SEM‐EDS analysis showed 0.36% of V only in BCAl + V in both warm and cold temperature experiments (Table S2; Figure S2). Adsorption parameters showed the highest *Q_M_* in BCAl at both temperatures, further complementing this result.

**FIGURE 4:**
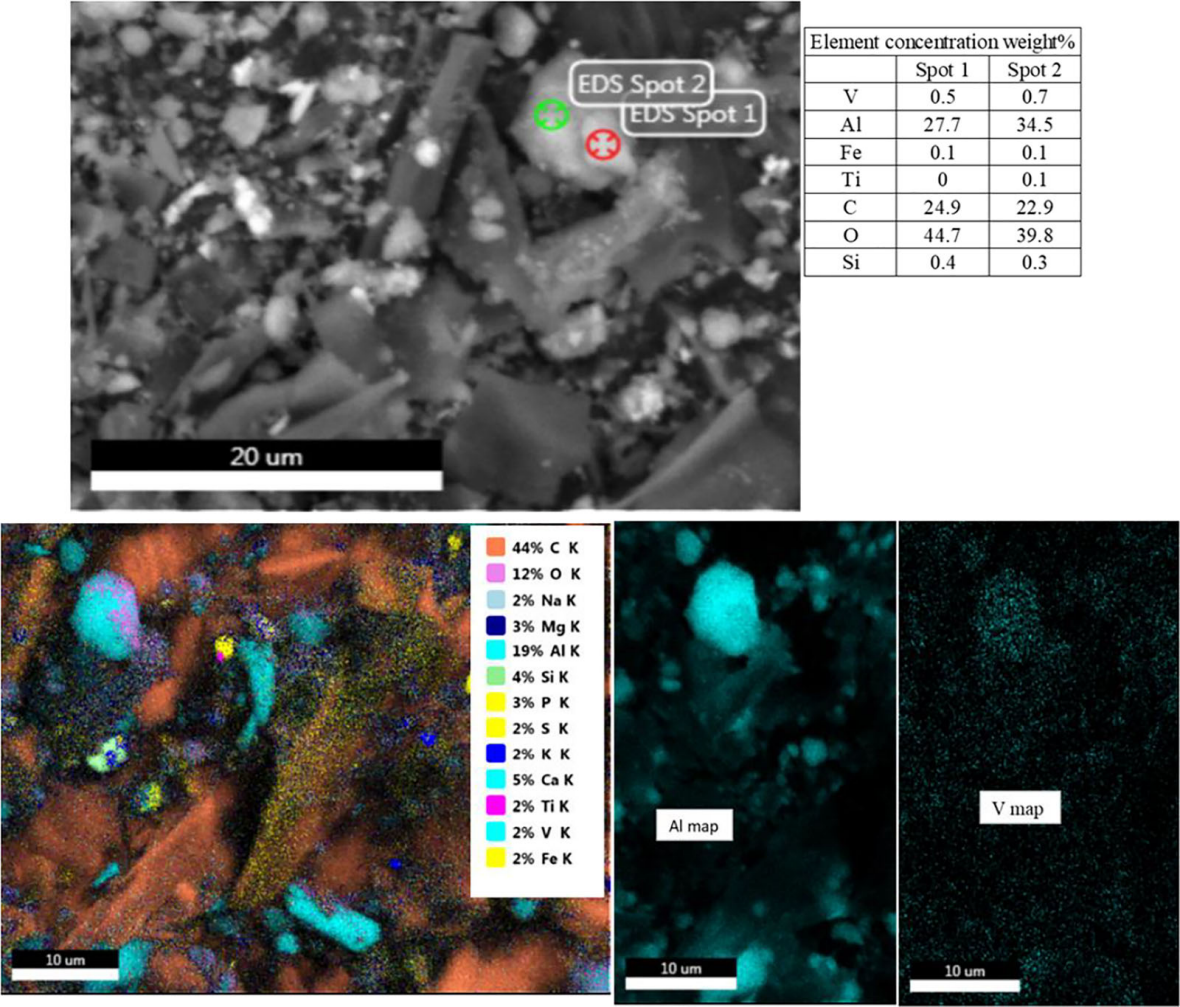
Secondary electron or backscattered electron (BSE) images collected at 15 keV at 2500 magnification, spot analysis of energy dispersive X‐ray spectra, EDS elemental overlay, and Al and V distribution in biochar‐Al oxide–V complexes.

The high‐magnification images and spot analysis with EDS showed a co‐existence of Al and V elements in BCAl–V complexes (Figure 4), suggesting V associations with Al on the surface layers. Overlapping of Al and V elements was further confirmed by the EDS distribution maps. Therefore, the SEM‐EDS results complement adsorption data by showing the highest adsorption in BCAl. Energy dispersive X‐ray spectra of BCAl also showed large amounts of O associated with V and Al (Figure 4). The co‐existence of Al, O, and V elements in surface layers of BCAl–V complexes signifies the bonding of vanadates to Al oxide surfaces.

**FIGURE 5:**
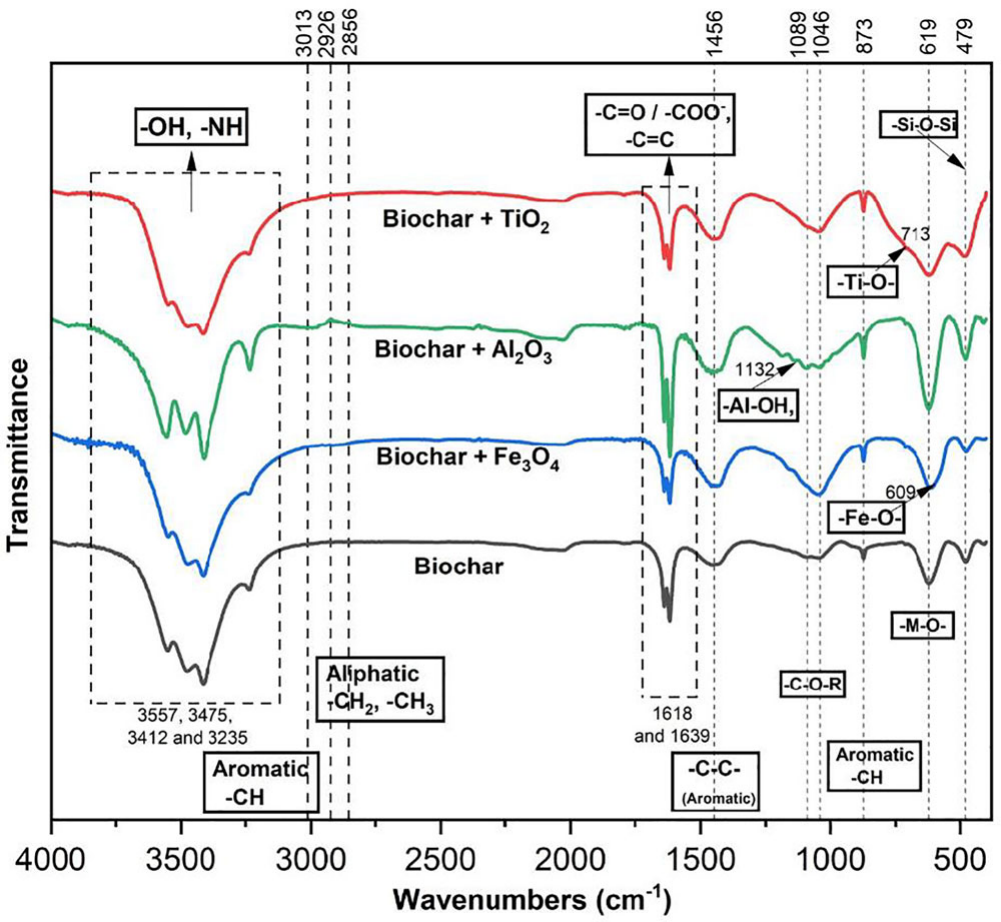
Fourier transform infrared patterns of biochar and biochar mixed with nano‐oxides of Fe_3_O_4_, Al_2_O_3_, and TiO_2_.

The FTIR spectra showed many different functional groups associated with BC and BC + metal oxide nanoparticle adsorbents, facilitating V retention onto those surfaces (Figure 5). The stretching and bending peaks of FTIR spectra revealed several functional groups in BC, BCFe, BCAl, and BCTi. Peaks observed in the region (3600‐3200 cm^−1^) could be attributed to O–H stretching from hydroxyl groups from chemisorbed water, phenols, or carboxyls (Jia et al., 2018; Q. Zhou et al., 2025). The peaks at 1618 and 1638 cm^−1^ are likely due to C═C stretching vibrations from aromatic rings and/or carboxylate (COO^−^), suggesting the presence of aromatic structures in the BC (Unuabonah et al., 2013; Wang et al., 2013; F. Zhang et al., 2016). The broadband at 1458 cm^−1^ can be attributed to C═C skeletal vibrations of aromatic rings, particularly from polycyclic aromatic structures in BC and/or C–H deformation (bending) vibrations of aliphatic (–CH_2_ and –CH_3_) groups in lignin (F. Zhang et al., 2016). Peaks at 1132–1160 cm^−1^ appeared in the BCAl, which could be due to Al–OH bonds indicating surface modification of the pure BC with Al nano‐oxides (T. Zhou et al., 2023). The peak at 1089 cm^−1^ could be linked with symmetric stretching vibration of –C–O–C– (glycosidic/ether bonds) and the peak at 1046 cm^−1^ due to –C–OH stretching. These peaks are characteristic of polysaccharides such as cellulose and hemicellulose (H. Chen et al., 2019; Inyinbor et al., 2016). The sharp peak at 873 cm^−1^ is associated with out‐of‐plane bending vibrations of the C–H bond in aromatics (C. Zhang et al., 2019). The broadband centered at 619 cm^−1^ is probably due to the combination of several metal‐oxygen (M–O) stretching (Jain & Gulati, 2023), and in this case, these could be Fe–O, Al–O, and Ti–O bonds. This M–O stretching peak was present in all amendments since the unmodified BC also contains ∼2% Al and Fe (Table S1). The peak at 479 cm^−1^ is due to the bending vibration of the silicon (Si)–O–Si bond (Qian et al., 2016). Therefore, FTIR peaks revealed the presence of –OH, C═C, C–H, –C–O–C, –C–OH, Al–O(H), Fe–O(H), Ti–O(H), and Si–O–Si functional groups or adsorption surfaces in BC and BC mixed with nano‐oxides of Fe, Al, and Ti.

## 4 DISCUSSION

### 4.1 BC and BC–metal oxide nanoparticle complexes as adsorbents for vanadium

The morphology of the amendments showed better microaggregates and smaller particle sizes in BCAl and BCTi compared to BC or BCFe. These morphological/structural changes also correlate with an increased adsorption capacity from 2528 to 4799 mg V kg^−1^, indicating high retention potential of BC and BC mixed with metal nano‐oxides. In previous studies, BC produced from wet‐distilled grain had the maximum sorption capacity of 1610 mg V(V) kg^−1^ compared to cow manure‐ and spent mushroom substrate‐BC (Kończyk et al., 2022). The V adsorption capacities of cesium‐modified, zinc‐modified, and zirconium‐modified‐BC were 3.22–5.55 times higher than that of commercial activated carbon (Meng et al., 2018). These results were related to their higher ion exchange capacities and specific surface areas of BC after modification. Although specific surface area was not directly measured in this study due to instrumentation limitations, SEM imaging revealed well‐developed microaggregates and porous structures in BC and BC–oxide composites, indicating a relatively high surface area that likely contributed to enhanced V adsorption. Organo‐mineral associations in BC facilitate pore formation at the interface of the carbon matrix and organo‐mineral aggregates (Archanjo et al., 2017). Adsorption mechanisms are influenced by key adsorbent characteristics, including surface area, particle size, pore structure, pore size distribution, and surface properties (Söğüt & Gülcan, 2023). Hence, microaggregation in BCAl and BCTi contributed to higher adsorption capacity compared to BC, and pore structure, pore size distribution, and surface properties may have contributed to high V adsorptions in all tested amendments in this study.

Previous studies reported the best fit of V adsorption with Ti and Fe oxides with the Langmuir model rather than the Freundlich where the adsorption study was conducted at a higher V concentration range of 5–250 mg L^−1^ (Meng et al., 2018; Naeem et al., 2007) in comparison to our study (0–40 mg L^−1^ V range). Our study isotherm data were fitted better to the Freundlich than the Langmuir, yet the conformity for both isotherms was significant. Hence, the isotherm parameters of both can be used for V adsorption interpretations. The Langmuir parameters, maximum adsorption capacity, and *Q_M_* showed the highest value in BCAl, followed by BCTi, while *K_L_* showed the highest value in BCTi followed by BCAl. According to Iftekhar et al. (2018), the Langmuir isotherm constant (*K_L_*) can be influenced by the pore volume‐to‐surface area ratio, which may explain the enhanced adsorption capacity observed in other modified adsorbents. While porosity and surface area were not measured in this study, our findings suggest that changes in surface characteristics (e.g., microaggregation) may have contributed to the observed variation in *K_L_* among treatments. Previous studies have reported V accumulation in the form of V(V) sorbed onto Fe and Al hydrous oxides in soils (Larsson et al., 2015), with strong V(V) sorption onto goethite reported by Peacock and Sherman (2004). The Freundlich parameter, *K_F_*, was highest in BCTi, followed by BCAl, while 1/*n* was lowest in BCTi, followed by BCAl. Therefore, both Freundlich and Langmuir parameters confirmed superior V adsorption in BC mixed with Al and Ti than in BC or BCFe.

The adsorption data conformed to both the Freundlich and Langmuir models, indicating that V adsorption involved a combination of chemisorption monolayer (Langmuir) and physisorption multilayer (Freundlich) mechanisms. The Langmuir model assumes monolayer adsorption of adsorbate on a homogeneous surface of adsorbent (Hu et al., 2011), whereas the Freundlich model accounts for heterogeneous adsorption, which is not restricted to a monolayer (Hu et al., 2011; Kim et al., 2013; Söğüt & Gülcan, 2023). The V adsorption data obtained for the BC and BC–metal nano‐oxide systems exhibited strong agreement with the Freundlich isotherm, suggesting adsorption onto a heterogeneous surface. The Langmuir model also provided a reasonable fit, suggesting the possibility of localized monolayer adsorption. However, spectral peak shifts in the mid‐infra red region were not observed with our instrument, which may indicate that there were minimal structural changes to the functional groups (C═O, C═C, C–O, O–H) as a result of V adsorption. While isotherm model fitting and the presence of functional groups support mechanisms such as surface complexation or chemisorption, confirmation of these interactions would benefit from complementary spectroscopic analyses (e.g., X‐ray photoelectron spectroscopy [XPS] or extended X‐ray absorption fine structure [EXAFS]) to resolve the nature of V binding more precisely. The effects of temperature, adsorbent type, and their interaction were significant for all adsorption parameters. However, the temperature effect was only significant in BCTi for *Q_M_* and *K_F_* in BCAl. The *Q_M_* of BCTi in cold temperature was higher than in warm temperature, while *K_L_* in BCAl was higher in warm temperature than in cold temperature, indicating inconsistencies in temperature effect on adsorption parameters. Vanadium speciation was predicted using Visual MINTEQ modeling, which indicated a shift from orthovanadate to polyvanadate species at 4°C compared to 22°C. Abernathy et al. (2022) reported H_2_VO_4_^−^ as the dominant V species at circumneutral pH. Due to steric hindrances, a decrease in adsorption of large molecules, that is, polyvanadates, can be expected. Although polyvanadate species with steric hindrance may reduce surface adsorption, BCTi exhibited higher adsorption at 4°C compared to 22°C. This contrasts with Kajjumba et al. (2018), who observed increased V adsorption with increasing temperature using shale and coal waste at pH 3, indicative of an endothermic chemisorption process. Our study, conducted at pH 7.5 with BC–nanoparticle composites, involves various V species and different sorption mechanisms. Similarly, Peqini et al. (2025) found that temperature effects on Cr(VI) and Cu(II) adsorption to BC–nanoparticle composites were minimal or nonexistent, supporting the view that temperature responses are highly dependent on the adsorbent composition and interaction mechanisms. In our case, temperature effects varied across BC, BCAl, BCTi, and BCFe, suggesting that the type of metal oxide incorporated plays a critical role in adsorption behavior.

Surface characteristics, as of the Freundlich parameters in BCTi, showed the highest heterogeneity under both warm and cold temperature conditions. Vessey et al. (2020) observed low affinity of polyvanadate species for ferrihydrite surfaces compared to that for hematite. Another study conducted for V(V) adsorption onto different mineral surfaces concluded that V(V) adsorption is exergonic for a variety of surfaces with differing amounts of terminal‐OH groups and metal–O bond saturations (Abernathy et al., 2021). These findings highlight the need to interpret temperature effects on V adsorption in relation to both composite composition and dominant sorption pathways, rather than applying generalized trends across materials.

### 4.2 Vanadium retention mechanisms onto BC and BC–metal oxide nanoparticle complexes

The adsorption mechanisms of heavy metals generally involve multiple interactions, including ion exchange, pore filling, surface precipitation, metal anion complexation, and electrostatic attraction (Tan et al., 2015). Due to the low detection limit of EDS (0.1% w/w or ∼1000 mg kg^−1^), only BCAl, which exhibited the highest V adsorption, showed detectable V concentrations on its surface. SEM‐EDS elemental mapping of BC produced through different methods revealed V compositions ranging from 0.0% to 0.6% (Kończyk et al., 2022). The co‐occurrence of Al, O, and V indicated that V retention occurred through surface complexation on the BCAl surface layers. Vanadium can strongly bind or sorb onto metal (hydr)oxides in soils, particularly Fe, Al, Ti, and manganese (Mn) (hydr)oxides, thereby significantly reducing its bioavailability (Haak et al., 2024; Larsson et al., 2017; Shaheen et al., 2019). While bioavailability was not directly assessed in this study, the strong sorption observed suggests a potential for reduced environmental risk, as supported by previous findings. Furthermore, V has been demonstrated to form covalent, inner‐sphere complexes with various mineral phases, reinforcing its strong adsorption behavior (Abernathy et al., 2021).

The chemical characteristics of BC surfaces are directly related to their composition, as evidenced by FTIR spectral peaks indicating the presence of –OH, C–H, C–H_2_, Si–O, C–O, Al–O(H), Fe–O(H), Ti–O(H), and Si–O–Si functional groups across all adsorbents, with minor variations. However, spectral peak shifts were not observed following V adsorption, indicating that we were unable to conclusively confirm specific binding mechanisms using FTIR in this study. The functional groups present in sorbent structures play a crucial role in the adsorption of V onto sorbent surfaces, and their identification provides insight into the nature of binding interactions between the sorbate and the sorbent (Iqbal et al., 2009). The adsorption properties of BC are largely influenced by its surface functional group composition (Pintor et al., 2012). FTIR analysis of BC derived from different organic materials has also revealed functional groups associated with O–H bending, carboxyl (C═O), alcoholic or carboxylic acid (C–O), C–H, O–H stretching, C–O–H, and Si–O–Si bending (Kończyk et al., 2022). The surface adsorption properties of BC surfaces are directly related to composition, as evidenced by FTIR spectral peaks indicating the presence of metal complexing groups such as alkenes (C═C) and oxygen‐containing groups (C–O; C–OH), resulting in the observation of Al–O(H), Fe–O(H), and Ti–O(H) in all of the BC‐metal nano‐oxide samples.

Since V exists as vanadate species such as H_2_VO_4_^−^ and HPO_4_^2−^, electrostatic interactions alone are unlikely to play a significant role on BC and nano‐oxide surfaces. Inner‐sphere V(V) complexes were detected on Fe and Mn mineral phases of ferrihydrite, hematite, goethite, birnessite, and pyrolusite through EXAFS and thermodynamic calculations (Abernathy et al., 2022). The high abundance of various O‐containing functional groups on the surfaces, along with the hydrophilic nature of the wood BC, contributed to high V immobility in BC‐amended soils (El‐Naggar et al., 2021; Weber & Quicker, 2018). The incorporation of nano‐oxides further enriched M‐O functional groups enhancing V(V) retention in BC–nano‐oxide mixtures. The formation of inner‐sphere complexes and bidentate‐mononuclear edge‐sharing complexes between V(V) and oxide surfaces (−OH and metal–O groups) in soils was also revealed previously (Abernathy et al., 2021; Vessey & Lindsay, 2020). Therefore, the interaction mechanisms between nano‐oxides and V are likely to involve a combination of electrostatic attraction, hydrogen bonding, and inner‐sphere complex formation between M–O groups and vanadate species. Although our FTIR results did not show distinct spectral changes after adsorption, the presence of key functional groups on the BC surface supports the plausibility of these mechanisms and is consistent with those reported in previous studies.

While this study demonstrates the adsorption efficiency and surface interactions of BC and nanoparticle composites with V, the long‐term stability of these complexes and their impact on V bioavailability in soil systems remain to be validated. These questions are currently being addressed in a follow‐up study involving field‐contaminated soils.

## 5 CONCLUSIONS

Vanadium is a re‐emerging contaminant of concern in both soil and water environments, requiring cost‐effective and environmentally compatible remediation strategies. This study evaluated the adsorption performance of wood‐derived BC and its composites with Fe, Al, and Ti nano‐oxides across temperature conditions representative of temperate and cold climates.

Adsorption isotherm modeling and vanadium speciation predictions showed that temperature influences vanadate forms and sorption behavior. Among the tested composites, the BCAl mixture exhibited the highest V adsorption capacity. SEM‐EDS analysis confirmed the presence of Al and V in BCAl–V complexes, while FTIR identified surface functional groups that may contribute to sorption; however, no significant spectral shifts were observed post‐adsorption.

While this study was limited to batch experiments and did not include field trials, the results highlight the influence of sorbent surface composition and temperature on V retention. These findings may inform future investigations into the development of composite materials for use in contaminated alkaline soils, particularly under variable climatic conditions. Further research is needed to evaluate long‐term stability, leaching behavior, and bioavailability of V in treated systems before practical applications can be considered.

## AUTHOR CONTRIBUTIONS

**Dileep Singh**: Data curation; formal analysis; investigation; methodology; writing—original draft; writing—review and editing. **Srimathie Indraratne**: Conceptualization; data curation; funding acquisition; project administration; resources; supervision; validation; visualization; writing—original draft; writing—review and editing. **Bhavya Anil**: Data curation; formal analysis; investigation; methodology; writing—review and editing. **Melissa Haak**: Formal analysis; investigation; methodology. **Darshani Kumaragamage**: Conceptualization; project administration; resources; visualization; writing—review and editing. **Doug Goltz**: Conceptualization; project administration; resources; supervision; writing—review and editing.

## CONFLICT OF INTEREST STATEMENT

The authors declare no conflicts of interest.

## Supporting information



The supplemental materials include tables of : (i) biochar elemental composition (Table S1), (ii) physicochemical properties of the nano‐oxides (Table S2), (iii) ANOVA results for Langmuir and Freundlich parameters (Table S3), and (iv) SEM‐EDS results of the amendments (Table S4). Two figures present (i) a SEM image of biochar (Figure S1), and (ii) SEM‐EDS mapping results (Figure S2). Additional methodological details were given too.
