# Supplementary material for: Temperature effects on vanadium speciation and adsorption to biochar alone and biochar–metal oxide nanoparticle composites
Source: J Environ Qual. 2026 Jan 22;55(1):e70139. doi: 10.1002/jeq2.70139 (PMC12827837; doi:10.1002/jeq2.70139)
Supplement: Supplementary file 1 — The supplemental materials include tables of : (i) biochar elemental composition (Table S1), (ii) physicochemical properties of the nano‐oxides (Table S2), (iii) ANOVA results for Langmuir and Freundlich parameters (Table S3), and (iv) SEM‐EDS results of the amendments (Table S4). Two figures present (i) a SEM image of biochar (Figure S1), and (ii) SEM‐EDS mapping results (Figure S2). Additional methodological details were given too. [file JEQ2-55-0-s001.docx]

**Temperature effects on vanadium speciation and adsorption to biochar alone and biochar-metal oxide nanoparticle composites**

Dileep Singh^1^, Srimathie Indraratne^*1^, Bhavya Anil^1^, Melissa Haak^1^, Doug Goltz^2^, Darshani Kumaragamage^1^

(1) Department of Environmental Studies and Sciences, The University of Winnipeg, Winnipeg, Manitoba, R3B 2E9, Canada

(2) Department of Chemistry, The University of Winnipeg, Winnipeg, Manitoba, R3B 2E9, Canada

(3) Department of Soil Science, University of Manitoba, Winnipeg, MB, Canada

Corresponding author: s.indraratne@uwinnipeg.ca

**Supplemental materials**

The supplemental materials include tables of : (i) biochar elemental composition, (ii) physicochemical properties of the nano-oxides, iii) ANOVA results, and (iv) SEM-EDS results of the amendments. Two figures present (i) a SEM image of biochar and (ii) SEM-EDS mapping results. Additional methodological details are also provided.

Methodology

All bottles and glassware were soaked for 24 h in 0.1% Alconox solution, triple rinsed with de-ionized water, and then submerged in a 10% nitric acid solution overnight. All glassware was then triple-rinsed with Milli-Q water and allowed to air-dry.

Sample digestion and total vanadium analysis were conducted by ALS Global Laboratories (Canada), following their standard 4-acid near-total digestion protocol.

Each sample (0.25 g) was digested using a mixture of concentrated HNO₃, HCl, HF, and HClO₄ in a closed-vessel microwave system, optimized for total metal extraction from soils and organic-rich materials. The digestion was performed using a closed-vessel microwave-assisted digestion system, as per ALS's certified in-house protocols. The residue was leached with hydrochloric acid (HCl) and diluted to 25 mL. The Al, Fe and Ti concentrations were determined by inductively coupled plasma-atomic emission spectroscopy (ICP-AES; , Agilent VISTA 725). The analytical results were corrected for interelement spectral interferences (ALS code ME-MS61).

Vanadium Analysis by ICP-OES

Vanadium concentrations in aqueous samples were measured using inductively coupled plasma optical emission spectroscopy (ICP-OES) with a Thermo iCAP 6500 Duo (Cambridge, UK) equipped with a duo-view axial/radial torch configuration and a Teledyne CETAC ASX-280 autosampler. All measurements were carried out in the axial torch configuration and V analysis was conducted using the 289.322 nm emission line, which showed the best sensitivity with minimal spectral interferences under the matrix used (0.01 M NaNO₃). To ensure there were no spectral interferences, 2 additional emission wavelengths (290.882nm and 292.402 nm) were also monitored.

Calibration curves were performed using a series of matrix-matched V standard solutions (0, 1, 10, and 40 mg/L) prepared from a 1000 mg/L single-element stock solution (Thermo Scientific). External calibration curves showed excellent linearity with an R² > 0.999. Quality control (QC) standards and were run every 15 samples to ensure analytical stability and a multi element QC sample containing V was included in each batch of samples to ensure accuracy.

All samples were filtered through 0.45 µm membranes prior to analysis and acidified to pH < 2 with ultrapure HNO₃ (Aristar Plus, VWR). Blanks and method standards were included in each run.

The limit of detection (LOD) and limit of quantification (LOQ) for vanadium were determined based on 3× and 10× the standard deviation of 10 replicate blank measurements, respectively. The calculated LOD was 3.54 µg/L and LOQ was 11.7 µg/L.

Analytical precision was assessed by triplicate measurement of selected samples, and relative standard deviations (RSDs) were typically <3%. Spike recovery tests performed on random samples showed recoveries between 95–104%, indicating satisfactory accuracy under the given matrix conditions.

**Results**

Table S1. Aluminum, iron, and titanium % of biochar after wet digestion method

| Element | Content % |
| --- | --- |
| Al | 2.12 |
| Fe | 1.90 |
| Ti | 0.26 |

Table S2: Physicochemical properties of nano-oxide materials used in the experiment.

| Product | Aluminum oxide | Iron(II,III) oxide | Titanium dioxide |
| --- | --- | --- | --- |
| CAS Number | 1344-28-1 | 1317-61-9 | 13463-67-7 |
| Chemical Formula | Al_2_O_3_ | Fe_3_O_4_ | TiO_2_ |
| Molecular Weight (g/mol) | 101.96 | 231.53 | 79.87 |
| Form | Nanopowder | Nanopowder | Nanopowder |
| Particle Size (TEM) | <50 nm | 50-100 nm | 21 nm |
| Surface Area (BET) m2/g | 40-60 | 40-60 | 35-65 |
| Density (g/cm³) | 3.95 | 5.17 | 4.23 |
| Supplier | Sigma-Aldrich | Sigma-Aldrich | Sigma-Aldrich |
|  |  |  |  |

Table S3. ANOVA results for Langmuir (Q_M_, K_L_) and Freundlich (1/n, K_F_) parameters

|  | ANOVA tabel |  |  |  |
| --- | --- | --- | --- | --- |
|  | Q_M_ | K_L_ | 1/n | K_F_ |
| Treatment effect | p <0.000 | p<0.000 | p<0.000 | p<0.000 |
| Temperature effect | p=0.003 | p=0.004 | p<0.000 | p<0.000 |
| Interaction effect | p=0.021 | p<0.000 | p<0.000 | p<0.000 |

Table S4. Mean of element concentrations (weight %) in the amendment-V complexes formed at warm (all amendments) and cold (BCAl only) temperatures subjected to SEM–EDS analysis (standard error of mean is given with in brackets, n=3)

| Element | BC+V 22°C | BCFe+V 22°C | BCAl+V | | BCTi+V 22°C |
| --- | --- | --- | --- | --- | --- |
|  |  |  | 22°C | 4°C |  |
| V | ND | ND | 0.36(±0.03) | 0.36(±0.03) | ND |
| Al | 0.73(±0.03) | 0.63(±0.03) | 10.63(±0.08) | 10.56(±0.14) | 0.56(±0.03) |
| Fe | ND | 16(±0.2) | 0.53(±0.05) | 0.53(±0.05) | 0.7(±0.1) |
| Ti | ND | ND | ND | ND | 16.2(±0.23) |
| O | 21.13(±0.12) | 22.13(±0.34) | 27.96(±0.46) | 29.83(±0.08) | 25.1(±0.35) |
| C | 70.86(±0.38) | 54.56(±0.08) | 59.46(±1.27) | 52.26(±0.14) | 50.86(±0.91) |
| Si | 1.53(±0.06) | 1.13(±0.03) | 1.13(±0.06) | 1.13(±0.06) | 1.14(±0.05) |


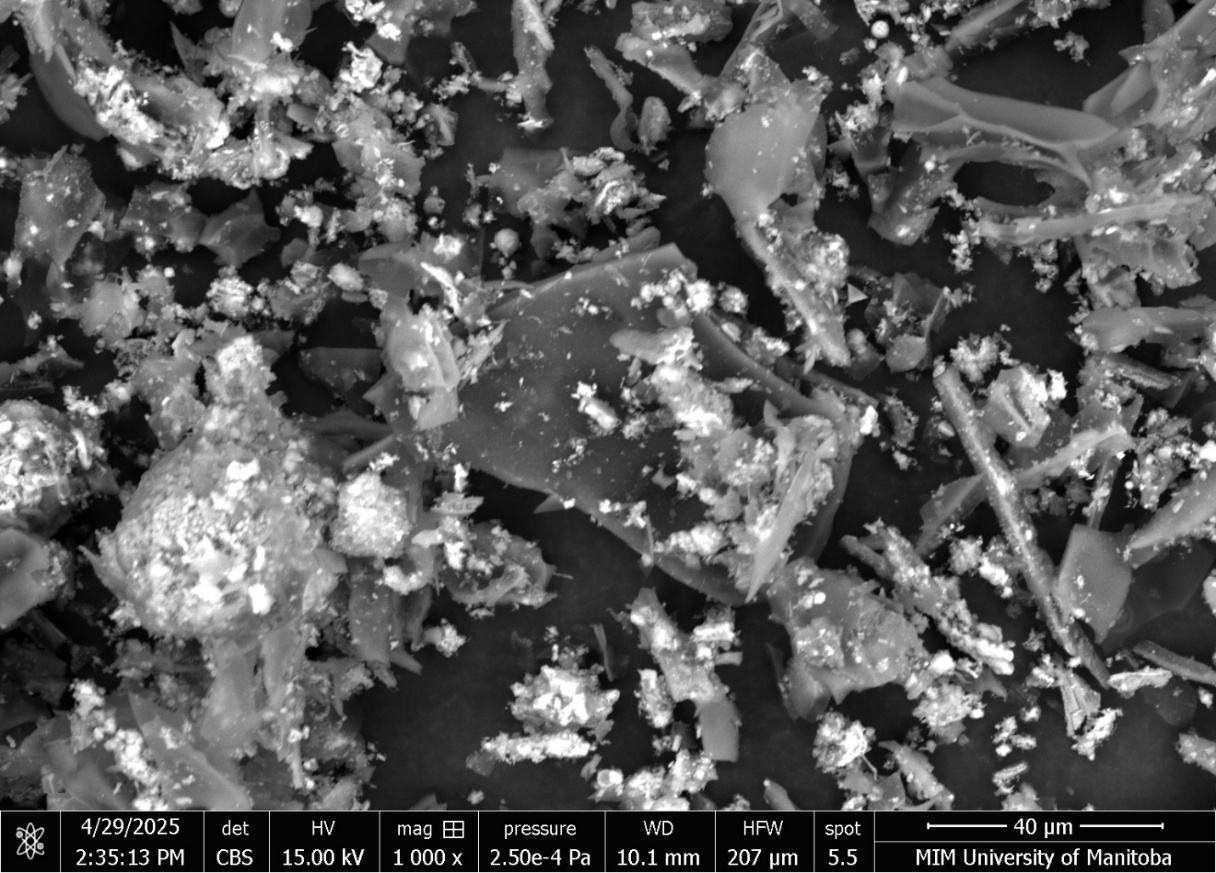


Figure S1. Secondary electron or backscattered electron (BSE) images collected at 15 keV at 1000 magnification for pristine biochar


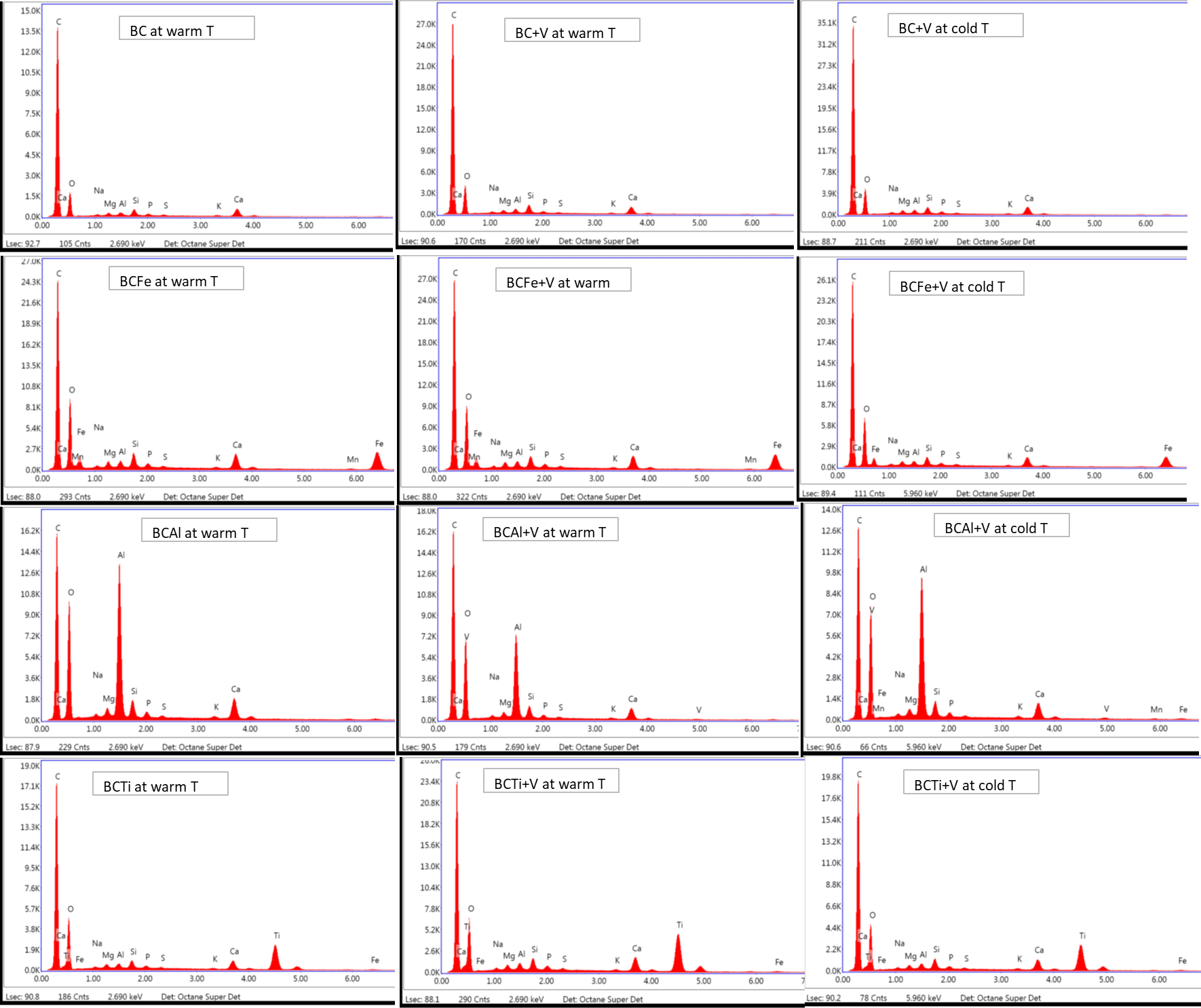


Figure S2. SEM-EDS elemental composition of amendment-V solid (amendments were equilibrated with V at 40 mg L^-1^
